# Sepsis and septic shock: endothelial molecular pathogenesis associated with vascular microthrombotic disease

**DOI:** 10.1186/s12959-019-0198-4

**Published:** 2019-05-30

**Authors:** Jae C. Chang

**Affiliations:** Department of Medicine, University of California Irvine School of Medicine, Irvine, CA USA

**Keywords:** Anti-microthrombotic therapy, C5b-9 (membrane attack complex [MAC]), Disseminated intravascular coagulation (“DIC”, ill-founded DIC), Disseminated intravascular microthrombosis (DIT), Endotheliopathy, Microthrombogenesis, Multiorgan dysfunction syndrome (MODS), Therapeutic plasma exchange (TPE), TTP-like syndrome, Unusually large von Willebrand factor multimers (ULVWF), Vascular microthrombotic disease (VMTD)

## Abstract

In addition to protective “immune response”, sepsis is characterized by destructive “endothelial response” of the host, leading to endotheliopathy and its molecular dysfunction. Complement activation generates membrane attack complex (MAC). MAC causes channel formation to the cell membrane of pathogen, leading to death of microorganisms. In the host, MAC also may induce channel formation to innocent bystander endothelial cells (ECs) and ECs cannot be protected. This provokes endotheliopathy, which activates two independent molecular pathways: inflammatory and microthrombotic. Activated inflammatory pathway promotes the release of inflammatory cytokines and triggers inflammation. Activated microthrombotic pathway mediates platelet activation and exocytosis of unusually large von Willebrand factor multimers (ULVWF) from ECs and initiates microthrombogenesis. Excessively released ULVWF become anchored to ECs as long elongated strings and recruit activated platelets to assemble platelet-ULVWF complexes and form “microthrombi”. These microthrombi strings trigger disseminated intravascular microthrombosis (DIT), which is the underlying pathology of endotheliopathy-associated vascular microthrombotic disease (EA-VMTD). Sepsis-induced endotheliopathy promotes inflammation and DIT. Inflammation produces inflammatory response and DIT orchestrates consumptive thrombocytopenia, microangiopathic hemolytic anemia, and multiorgan dysfunction syndrome (MODS). Systemic inflammatory response syndrome (SIRS) is a combined phenotype of inflammation and endotheliopathy-associated (EA)-VMTD. Successful therapeutic design for sepsis can be achieved by counteracting the pathologic microthrombogenesis.

## Background

**Fig. 1:**
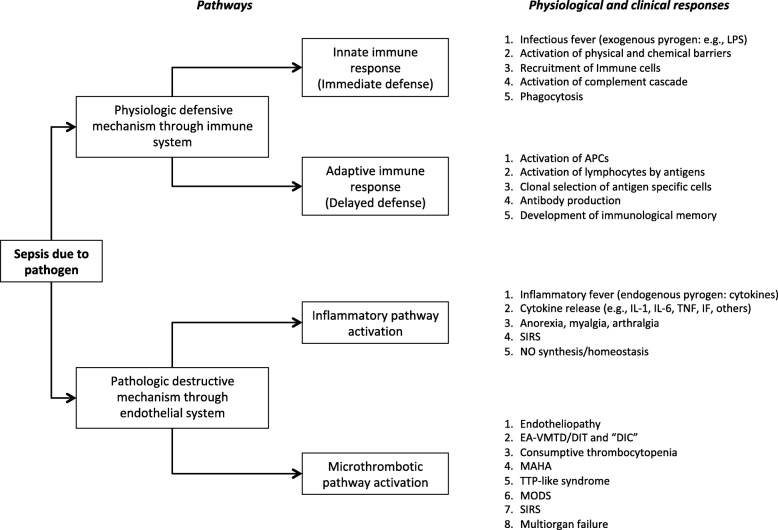
Physiological and pathological response mechanisms in sepsis. In sepsis, host response is characterized by two mechanisms. One is physiologic defensive mechanism through immune system, and the other is pathologic destructive mechanism through endothelial system. The physiologic response and pathologic clinical syndromes are notated in the Figure. Now, we know that complement system, protecting the host through innate immune system, could trigger harmful endothelial pathogenesis. This dual role of the complement must be nature’s rule just like normal hemostasis, which protects human lives in external bodily injury, but also may harm human lives in intravascular injury through thrombogenesis. Abbreviations: *APC* antigen presenting cell, “*DIC*” disseminated intravascular coagulation, *DIT* disseminated intravascular microthrombosis, *EA-VMTD* endotheliopathy-associated vascular microthrombotic disease, *MAHA* microangiopathic hemolytic anemia, *MODS* multiorgan dysfunction syndrome, *MOF* multiorgan failure, *NO* nitric oxide, *IF* interferon, *IL* interleukin, *LPS* lipopolysaccharide, *SIRS* systemic inflammatory response syndrome, *TNF* tumor necrosis factor, *TTP* thrombotic thrombocytopenic purpura

Sepsis is a serious life-threatening systemic physical illness associated with toxicity due to invasion of the bloodstream by pathogen. Invading pathogen can be bacteria, viruses, rickettsia, fungi or parasites. Early clinical manifestations begin with inflammation and progress to circulatory organ dysfunction associated with significant hematopathologic changes. Well-defined clinical phenotypic features include consumptive thrombocytopenia [1, 2], hemolytic anemia [1], vascular microthrombosis [1–4], multiorgan dysfunction syndrome (MODS) [3], coagulopathy [5] and septic shock. While natural defensive mechanisms of innate and adaptive immune systems of the host (Fig. 1) and effective antimicrobial therapy can favorably influence the course of sepsis, it is still accountable for roughly 15% of in-hospital deaths and 6.2% of discharges to hospice [6].

More recently, pathologic destructive mechanism through endothelial system (Fig. 1) has been suspected to be detrimental to the recovery of septic patient and contribute to the high morbidity and mortality [7–10]. Although the role of the endothelium in the pathogenesis of sepsis is not clearly established yet, coagulopathy has been proposed to play a key role through crosstalk mechanism between inflammation and coagulation as a result of systemic endothelial injury [11–13]. This theory has been particularly attractive because disseminated intravascular coagulation (“DIC”) commonly occurs in sepsis [14–16]. However, a quotation mark has been placed on “DIC” to note that it has been founded on the basis of poorly established concept of hemostasis [1–4]. Until recent controversy, “DIC” had been claimed to occur due to uncontrolled systemic activation of coagulation through tissue factor (TF)/FVIIa-initiated hemostasis, manifesting with thrombocytopenia, consumption coagulopathy, microvascular thrombosis and MODS [17, 18].

Now, a recurring question has come to light about the true character of “DIC”. Although it is a hemostatic disease, “DIC” is defined as microthrombotic disease different from macrothrombotic disease seen in arterial thrombosis and deep vein thrombosis [1–4]. This author has made a reinterpretation of “DIC” utilizing clinical, pathological, pathophysiological characteristics and molecular pathogenesis, and has determined it to be identical to disseminated intravascular microthrombosis (DIT) seen in thrombotic thrombocytopenic purpura (TTP)-like syndrome in recent publications [1–4, 19]. For the time being, both terms “DIC” and ill-founded DIC will be used interchangeably throughout this article.

## DIC and DIT in hemostasis and sepsis

**Fig. 2:**
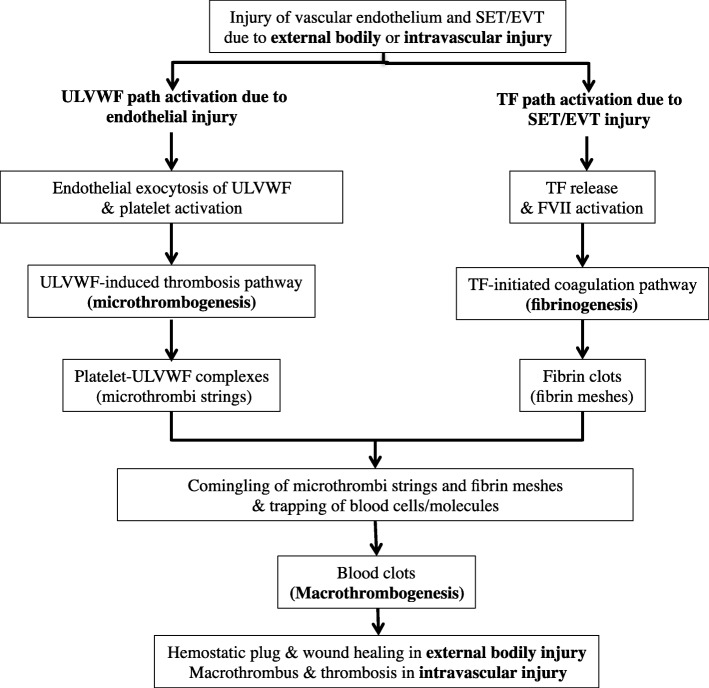
Three different paths of thrombogenesis that can occur within normal hemostasis. (Reproduced and updated with permission from Chang JC. Blood Coagul Fibrinoplysis. 2018; 29:573–84). Two different thrombotic paths, microthrombotic (ULVWF) and fibrinogenic (TF), are initiated in normal hemostasis, but later the two paths must unify to conclude normal hemostasis with passive role of NETs; it stops the bleeding in external bodily injury and produce the thrombosis in intravascular injury. However, in the different level (depth) of intravascular injury, thrombogenesis takes two different paths. If the level of intravascular injury is confined to the endothelium, lone ULVWF path become activated and causes microthrombosis (i.e., VMTD) because TF path is not activated. On the other hand, if the level of intravascular injury extends from the endothelium to SET/EVT, TF path becomes also activated and causes macrothrombosis (e.g., DVT). In one theoretical situation, if only SET/EVT is injured, available TF is supposed to activate TF path, but in reality this injury does not cause thrombosis without breached endothelium. However, in pathologic hemostasis, aberrant TF activation occurs and produces fibrin clots (i.e., true DIC) in APL due to TF expression in intravascular space from leukemic promyelocytes. APL is a consumption coagulopathy due to lone activation of TF path. This logic is based on “two-path unifying theory”. Please see Figure 2, showing 3 different thrombosis disorders via microthrombogenesis, fibrinogenesis, macrothrombogenesis, which are annotated in bold face. Each thrombognesis occurs when ULVWF path, TF path or combined paths are activated depending upon the levels of damage in intravascular injury (endothelium and SET/EVT). The characters of microthrombi, fibrin clots and macrothrombus from different paths are very different and produce distinctly different clinical thrombotic disorders [20]. Abbreviations: *APL* acute promyelocytic leukemia, *DIC* disseminated intravascular coagulation, *DVT* deep vein thrombosis, *EVT* extravascular tissue, *NET* neutrophil extracellular traps, *SET* subendothelial tissue, *TF* tissue factor, *ULVWF* unusually large von Willebrand factor multimers, *VMTD* vascular microthrombotic disease

**Fig. 3:**
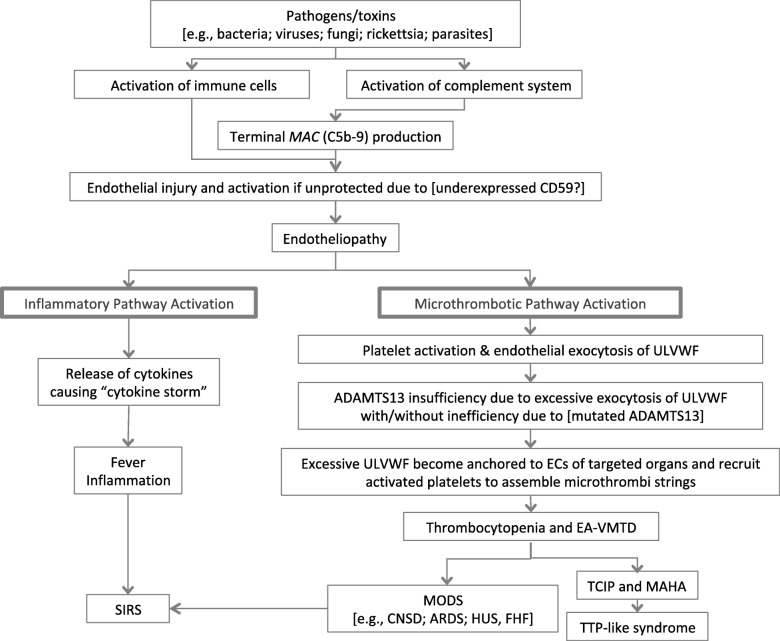
Endothelial molecular pathogenesis and microthrombogenesis in sepsis. (Based on “two-activation theory of the endothelium”). (Reproduced and modified from Thrombosis Journal 2018;16:20). Endothelial molecular pathogenesis is succinctly illustrated. Endotheliopathy activates two main pathways. Activation of inflammatory pathway produces cytokines, which main function is the modulation of inflammation, including fever and myalgia. Activation of microthrombotic pathway causes much more deadly septic syndromes via generalized EA-VMTD/DIT. Abbreviations: *CNSD* central nervous system dysfunction, *DIC* disseminated intravascular coagulation, *DIT* disseminated intravascular microthrombosis, *EA-VMTD* endotheliopathy-associated VMTD, *FHF* fulminant hepatic failure, *MAHA* microangiopathic hemolytic anemia, *SIRS* systemic inflammatory response syndrome, *TCIP* thrombocytopenia in critically ill patients, *TTP* thrombotic thrombocytopenic purpura, *ULVWF* unusually large von Willebrand factor multimers, * cell-mediated immune cells are T lymphocyte, macrophage, monocyte, neutrophil, and dendritic cell

To embrace the genuine characters between “DIC” and DIT, the pathophysiological mechanism of true DIC, “DIC” and DIT occurring as hemostasis should be clearly established. Indeed, through the better understanding of sepsis-associated coagulopathy, this has become possible to construct “two-path unifying theory” of hemostasis derived from the insights of endothelial pathogenesis that leads to microthrombogenesis and TTP-like syndrome. This novel theory, which is simple and easily understandable, was proposed and published [20] and updated in Fig. 2. This effort has allowed us to use the term disseminated intravascular microthrombosis (DIT) instead of “DIC” and endotheliopathy-associated vascular microthrombotic disease (EA-VMTD/DIT) as a distinct disease entity that is associated with TTP-like syndrome [1–4, 19, 20]. This new identity of “DIC” could provide a paradigm shift in the management of sepsis by utilizing targeted therapies to improve the outcome. In addition, this thesis along with “two-activation theory of the endothelium” (Fig. 3) identifies the true character of “DIC” [1–4, 20] as well as the mechanisms of thrombogenesis [4] and different phenotypes of the thrombotic disorder [3, 4, 20].

The hemostasis in sepsis-induced endotheliopathy has been poorly understood even though research scientists have known that endothelial injury plays a prominent role in sepsis and many other critical illnesses such as trauma, pregnancy, autoimmune disease, cancer and drug/toxin, leading to poorly defined but serious coagulopathy. The frequently noticed early hematologic manifestation is occurrence of unexplained “thrombocytopenia in critically ill patients” (TCIP), which until recently has been a clinical mystery [1, 2]. Now, it has become evident that thrombocytopenia develops due to contribution of platelets in microthrombogenesis and their consumption. Microthrombogenesis is the process of forming microthrombi composed of platelet-unusually large von Willebrand factor (ULVWF) complexes following platelet activation and endothelial exocytosis of ULVWF as a result of endotheliopathy [1–3].

EA-VMTD/DIT in sepsis is a unique hemostatic disorder developing via lone activation of ULVWF path without simultaneous activation of TF path according to “two-path unifying theory of hemostasis” [4, 20]. In this article, the pathophysiological mechanism promoting EA-VMTD/DIT in sepsis will be reviewed; the molecular pathogenesis of endotheliopathy and hemostasis will be discussed and the clinical phenotypes of various septic syndromes be analyzed. Finally, we should be able to recognize that EA-VMTD/DIT is the underlying clinical disease associated with sepsis-associated coagulopathy and oftentimes presents with hematologic phenotype of TTP-like syndrome.

## Endothelial pathogenetic mechanisms involved in sepsis

### Activation of complement system

The activation of complement system is normally one of the key events in defensive mechanism against pathogen in sepsis. Its protective function for the host rapidly identifies and eliminates invading pathogen whenever possible. Opsonization of foreign surfaces by covalently attached C3b fulfills three major functions: pathogen clearance by phagocytosis; amplification of complement activation by the formation of surface-bound C3 convertase; and assembly of C5 convertase. Cleavage of C5 induces the formation of the multi-protein pore complex C5b-9 (i.e., membrane attack complex [MAC]), which leads to lysis of pathogen [21].

Although the major role of complement is protective function for the host through innate immune defense, activated complement could also cause the destructive action to the host endothelium [22], which impacts the course of sepsis as illustrated in Fig. 1. MAC exerts harmful effects to host’s endothelial cells (ECs) [22] unless complement regulator CD59 is adequately expressed in ECs and protects them by inhibiting C9 polymerization into MAC [23, 24].

If CD59 is downregulated in the ECs due to either gene mutation or acquired diseases [25], activated terminal complement MAC could exert destructive effects to ECs in sepsis, trauma and other critical illnesses [26–28]. When MAC attacks innocent bystander ECs, channel (transmembrane pores) formation could occur on the endothelial membrane [22] and trigger endothelial dysfunction [3, 22, 29].

### The central role of the endothelium

The endothelium, located at the interface between blood and subendothelial tissue (SET)/ extravascular tissue (EVT), is a monolayer of endothelial cells distributed in the vasculature and organ system of the entire human body. Its vital function is maintaining not only homeostasis of circulatory networks but also providing hemostasis in vascular injury. It is essential to preserve both anatomical and functional integrity of the endothelium at all cost to prevent unneeded intravascular thrombosis from the exposure to TF present in SET/EVT [30], which could cause macrothrombosis and get involved in vascular and organ damage [20, 31].

Hemostasis plays a critical role in the pathogenesis of sepsis. Sepsis occurring due to a variety of pathogen causes generalized endothelial injury as a result of disseminated nature of the circulatory system, and leads to systemic endotheliopathy. However, sepsis-induced endotheliopathy triggers functional changes contributing to molecular dysfunction and subsequent partial hemostasis, mediating microthrombogenesis. Unlike localized traumatic intravascular injury, which initiates bleeding and hemostasis due to combined endothelial and SET/EVT damage [4, 20], anatomic disruption to the vascular endothelium is minimal in sepsis and bleeding is not consequential because endotheliopathy is confined to the endothelium and TF is not exposed [32]. Thus, complement activation due to pathogen or endotoxin provokes endothelial dysfunction promoting exocytosis of ULVWF and platelet activation, which initiates ULVWF path of hemostasis and leads to microthrombogenesis [2, 3] as shown in Figs. 2 and 3. This is a very important conception to understand sepsis-associated coagulopathy because sepsis promotes lone activation of ULVWF path without activation of TF path since SET/EVT damage does not occur. This thesis based on microthrombogenesis refutes the current theory that sepsis-associated “DIC” occurs via activation of TF path that leads to fibrinogenesis. Now, “DIC” is reinterpreted to be DIT that occurs via activated ULVWF-initiated thrombogenesis associated with endotheliopathy as illustrated in Fig. 2 [2–4, 20].

“DIC” is still the result of hemostasis even though TF is not involved [4, 20]. The exocytosis of ULVWF from Weibel-Palade bodies activates ULVWF path and platelets are easily recruited because ULVWF show extremely high affinity to the platelet. This lone activation of ULVWF path in endotheliopathy promotes the formation of microthrombi and lead to EA-VMTD/DIT [2, 3, 20]. Thus, microthombi of “DIC” are the same as those of TTP and TTP-like syndrome. “DIC” has been inappropriately conceptualized as a fibrin clot disease produced via activated TF/FVIIa-initiated cascade/cell-based coagulation. This ill-founded DIC must be properly renamed as EA-VMTD/DIT, which hematologic phenotype is TTP-like syndrome. On the other hand, consumption coagulopathy in acute promyelocytic leukemia (APL) that occurs due to pathologic activation of aberrant TF path caused by TF released from leukemic promyelocytes should be called true DIC [20]. True DIC in APL is made of disseminated fibrin clots that occur without vascular injury. Its hematologic phenotype is always characterized by hemorrhagic syndrome [2–4, 20].

### Endotheliopathy-associated microthrombosis as the crux of the clinical phenotypes

A hallmark of advancing sepsis is microvascular dysfunction [1, 8, 9] due to disseminated microthrombi composed of platelet-ULVWF complexes in multiorgans, which is promoted via microthrombogenesis [1, 2]. The pathophysiological mechanism inducing circulatory dysfunction that leads to organ ischemia has been well defined in clinical medicine as vascular microthrombosis [33, 34] or VMTD [1–4].

In addition to hypoxic microvascular dysfunction, endotheliopathy causes the release of various inflammatory cytokines [7, 35, 36] and bioactive biomarkers from ECs when infected by different types of pathogen [37]. Unlike adaptive immune mechanism that produces antigen-specific protective antibody response from each pathogen to each host, sepsis-induced endotheliopathy triggers similar, if not the same, endothelial molecular response without specificity among different types of pathogen (Fig. 3 and Table 1). This endothelial dysfunction causes independent inflammation different from vascular microthrombosis. The manifestations of inflammation and microthrombosis are universally similar among each class and each type of pathogens and display no specificity.

**Table 1:** Examples of thrombocytopenia (TCIP) in sepsis-associated coagulopathy (VMTD)

	Specific pathogens	Commonly involved organs	Associated clinical syndromes
Bacteria	Neisseria meningitides	adrenals; meninges	Waterhouse-Friderichsen syndrome
	*E. coli* O157:H7	bowels; kidneys; brain	GE; HUS; MODS
	MRSA	lung; multiorgans	ARDS
	Klebsiella pneumonia	lungs; multiorgans	ARDS; TAMOF
	Various bacterial sepsis	multiorgans	
Viruses	Ebola	lungs; liver; multiorgans	ARDS; hepatic necrosis
	H1N1 influenza	brain; lungs; multiorgans	Encephalopathy; ARDS
	MERS-CoV	lungs	ARDS
	SARS-CoV	lungs	ARDS
	Hantavirus	heart; lungs; kidneys	HCPS; HPS; HFRS
	Dengue	adrenals; multiorgans	DSS
	SFTS virus	multiorgans	SFTS
	Hepatitis A virus	liver	Fulminant hepatic failure
Fungi	*Candida albicans*	multiorgans	ARDS
Rickettsia	Rickettsia rickettsii	skin; multiorgans	RMSF
Parasites	Plasmodium falciparum	brain; multiorgans	Cerebral malaria; ARDS
	*Plasmodium vivax*	lungs; multiorgans	ARDS

*Abbreviations*: *ARDS* acute respiratory distress syndrome, *DSS* dengue shock syndrome, *GE* gastroenteritis, *HUS* hemolytic uremic syndrome, *MERS-CoV* Middle East respiratory syndrome-corona virus, *MODS* multiorgan dysfunction syndrome, *MRSA* methicillin resistant *Staphylococcus aureus*, *SARS-CoV* severe acute respiratory syndrome-corona virus, *SFTS* severe fever with thrombocytopenia syndrome, *TAMOF* thrombocytopenia and multiple organ failure, *HCPS* hantavirus cardiopulmonary syndrome, *HPS* hantavirus pulmonary syndrome, *HFRS* hemorrhagic fever with renal syndrome, *DSS* dengue shock syndrome, *RMSF* Rocky mountain spotted fever

However, an intriguing question is why then the clinical organ phenotype expression of sepsis is so different among the different hosts and also among the different pathogens. For example, the organ phenotypic features of sepsis could be encephalopathy, myocarditis, pancreatitis, acute respiratory distress syndrome, adrenal insufficiency, fulminant hepatic failure, hemolytic-uremic syndrome (HUS) and others, or any combination of them. This is not due to different kinds of VMTD, but is due to at least two underlying genetic variables governing individual organ susceptibility of the host and affinity of the pathogen to specific organ(s).

### Endothelial heterogeneity and organotropism

In endothelial pathogenesis of sepsis, the organ phenotype expression is variable among different hosts by the same pathogen as well as different pathogens. Organ phenotypes are determined by two main endowed biological mechanisms: endothelial heterogeneity of host [38, 39], and organotropism of pathogen [40–43]. Variable clinical organ phenotypic syndromes occur as seen in the same type of pathogen. Examples are hanta virus, causing cardio-pulmonary syndrome in the heart and lungs, Shiga toxin-producing *E. coli*, presenting with HUS (STEC-HUS) in the brain, bowels and kidneys, and Neisseria meningitides, inciting Waterhouse-Friderichsen syndrome and meningitis in the adrenals and meninges. Also, the same organ phenotype can occur in different types of pathogen.

Clinicians have used the mysterious combined clinical terms such as hepato-renal syndrome [44], hepatic encephalopathy [45], and cardio-pulmonary syndrome [46] often without identifying the involved pathogen even though sepsis might have existed. Perhaps, this oversimplified designation of organ phenotypes has impeded detecting the underlying etiology of organ syndromes. In modern medical literature, for example, many authors claimed one organ phenotypic syndrome causes several other organ dysfunctions such as acute renal failure, encephalopathy, and hepatic failure [47–49]. In sepsis, the concept of endothelial heterogeneity and organotropism for organ localization and the phenotype of VMTD as expression of underlying pathology support that biorgan or multiorgan manifestations are just different phenotypes of the same disease EA-VMTD [29]. Likewise, additional organ phenotypes that are often designated as extra-organ manifestations (e.g., extra-renal phenotypes in HUS, extra-pancreatic phenotypes in pancreatitis and extra-pulmonary phenotypes in acute respiratory distress syndrome [ARDS]) are expression of the same hemostatic disease EA-VMTD. Indeed, extra-organ phenotypes are misrepresentation.

The organ phenotype expression associated with EA-VMTD is determined by combined mechanism of underlying endothelial heterogeneity of each host [29, 38, 39] and organ/tissue tropism of each pathogen/toxin [40]. For examples, the phenotypes of STEC-HUS [29, 41] are the combined results of genotypic endothelial heterogeneity of the host and genotypic tropism of STEC on the gastrointestinal tracts, kidneys, lungs, brain and others. Likewise, the phenotypes of Waterhouse-Friderichsen syndrome [50] associated with Neisseria meningitides are expressed as a result of combined endothelial heterogeneity of the host and tissue tropism of the bacteria in the adrenals and meninges. These concepts are very important in the understanding of genesis of MODS.

### Molecular pathogenesis based on “two-activation theory of the endothelium”

Although endotheliopathy is proposed to be the main pathology promoting crosstalk mechanism between inflammation and coagulation in sepsis, both the character of blood clots and thrombogenetic mechanism producing coagulopathy could not be comprehensible by this crosstalk theory [4]. However, because complement activation in critical illnesses affects several molecular functions of ECs, it fits well with endothelial pathogenesis of sepsis.

The “two-activation theory of the endothelium” (Fig. 3) [1–4, 19, 20] has been proposed based on the role of endotheliopathy not only in sepsis, but also in other critical illnesses. Once endotheliopathy occurs in sepsis, endothelial dysfunction promotes the activation of two independent endothelial pathways; one is inflammatory and the other is microthrombotic. In short, two main molecular events are: 1) release of inflammatory cytokines such as interleukin (IL)-1, IL-6, tumor necrosis factor-α, and others [1–3, 7, 35, 36], and 2) activation of the platelet [51, 52] and exocytosis of ULVWF [53, 54]. The former triggers inflammation through “activated inflammatory pathway”, and the latter mediates microthrombogenesis via “activated microthrombotic pathway”. Inflammation promotes inflammatory symptoms, but microthrombogenesis mediates thrombosis to produce microthrombi strings composed of platelet-ULVWF complexes [54–56]. Micothrombogenesis is activated when the protease ADAMTS13 is insufficient to cleave the excess of exocytosed ULVWF with or without mild to moderate underlying enzyme deficiency that occurs due to polymorphism or heterozygous mutation of ADAMTS13 [57–60]. These microthrombi strings produce DIT in the smaller and larger vasculatures of various organs [1–4].

### Mechanism of sepsis-associated coagulopathy

According to novel “two-path unifying theory” (Fig. 2), in intravascular injury normal hemostasis begins with simultaneous but independent activation of ULVWF path and TF path [4, 20]. However, in endotheliopathy associated with sepsis, only ULVWF path of normal hemostasis becomes activated because TF is not available in the endothelium [32]. Endotheliopathy in sepsis triggers exocytosis of ULVWF and activates platelets, and produces microthrombi strings. The microthrombi strings composed of platelet-ULVWF complexes must be the intrinsic character of the blood clots in “DIC” of sepsis that occurs as the result of lone activation of ULVWF path illustrated in Fig. 2. Therefore, the concept of “DIC” in sepsis-associated coagulopathy is due to activated TF path producing “fibrin clots” is an incorrect interpretation. This interpretative mistake has been very costly. Thus, according to “two-path unifying theory”, “DIC” is the same to EA-VMTD/DIT and acute “DIC” is EA-VMTD/DIT with hepatic coagulopathy/hemorrhagic syndrome. EA-VMTD/DIT without hemorrhagic syndrome had been called chronic “DIC” as previously detailed [2–4, 20].

The endothelium positioned at the interface between the blood and SET/EVT functions as the initiating site of the foundry producing thrombus following vascular injury [4]. Because sepsis-induced vascular injury is typically limited to the endothelium, only ULVWF path becomes activated and causes vascular microthrombosis in the smaller and larger vasculatures [3]. Sepsis-associated coagulopathy has shown to express the changes in antithrombotic and prothrombotic markers, which include TF path-related markers such as thrombin, antithrombin, thrombin-antithrombin complexes, activated protein C (APC), tissue factor pathway inhibitor (TFPI), and thrombomodulin (TM). These expressed TF path markers have indirectly supported the role of activated TF path in the pathogenesis of “DIC”. However, these TF path-related markers can be explained by alternative mechanisms, including acute DIC with hepatic coagulopathy, combined micro-macrothrombotic syndrome, concomitant vascular injury as a result of surgery and vascular access, and perhaps more commonly MODS resulting in tissue necrosis, especially of the liver, kidneys and lungs, in advancing severe sepsis. In retrospect, expressed TF path markers in sepsis can be ascribed to secondary events that are unrelated to primary pathogenesis of activated ULVWF path as illustrated in Fig. 2.

In patients with severe sepsis, thrombin generation, APC, TFPI and TM are not the true markers of “DIC”, but are interpreted as secondary markers of TF path. The true markers of “DIC” are activated platelet, increased FVIII activity, and increased VWF from, excessive release of ULVWF, and upregulated collagen binding that result from activated ULVWF path [4, 20]. It is no wonder why clinical trials using antithrombin agents, APC, recombinant TFPI and recombinant TM was unsuccssful in sepsis-associated coagulopathy. It was likely due to the fact that sepsis-associated “DIC” is not true DIC, but is EA-VMTD (i.e., TTP-like syndrome) caused by activated ULVWF path. Further, endothelial injury alone cannot induce fibrinogenesis, but only provokes partial hemostasis, leading to microthrombogenesis, and produces EA-VMTD. On the other hand, APL-associated DIC that occurs due to activation of aberrant TF path is true DIC [20].

In sepsis-associated coagulopathy, this author believes neutrophil extracellular traps (NETs) participate in thrombosis in the unifying stage of macrothrombus, but the problem of NETosis is with the logic of hemostatic principle and inconsistent characters of thrombosis in vivo and animal models in the reported literature. The essential logic in the formation of thrombosis is normal hemostasis must be accompanied by intravascular injury [4, 20]. Without it, no thrombosis can be formed because activation of ULVWF path and/or TF path is sine qua non in thrombogenesis. Further, only three exceptions among thrombotic disorders occur without vascular injury. They are (1) TTP, which is aberrant thrombosis because vessel is not injured, but still utilizes “ULVWF” path in ADAMTS13 deficiency, (2) APL, which is aberrant thrombosis because vessel is not injured, but still utilizes “TF” path due to overexpressed TF from leukemic promyelocytes, and (3) heparin-induced thrombocytopenia with thrombosis syndrome, which is pathologic thrombosis because vessel is not injured, and also utilizes neither ULVWF path nor TF path [4, 20]. However, we know cellular and molecular traps, and perhaps adhesion molecules participate in the hemostatic plug and thrombus formation, which is illustrated in Fig. 2, where blood cells/molecules are trapped in comingling process during blood clots formation (macrothrombosis) in the unifying stage of microthrombi strings and fibrin clots. This author believes that NETosis is not active hemostatic processes, but is a passive one associated with secondary event trapping blood cells and molecules such as DNAs and histones in the process of thrombogenesis.

Now, supported by “two-activation theory of the endothelium” and “two-path unifying theory of hemostasis” in the understanding of sepsis and septic shock, we can easily define all clinical phenotypes of the sepsis-associated syndromes via two independent endothelial pathways promoting inflammation and microthrombogenesis.

## Sepsis-associated clinical syndromes

### Inflammation

Inflammation is a clinical phenotype of the endothelial pathogenesis in sepsis, which presents with inflammatory symptoms such as fever, chills, headache, myalgia and arthralgia. Inflammatory fever occurs due to endogenous pyrogens (e.g., cytokines IL-1, IL-6, TNF, and others) and is different from infectious fever, which occurs due to exogenous pyrogens (e.g., lipopolysaccharide) [61–63] as physiologic defensive mechanism through immune system as shown in Fig. 1. Clearly there are two kinds of fever combined in sepsis. One is infectious fever due to exogenous pyrogen and the other is inflammatory fever caused by endotheliopathy [61, 62]. In sepsis, fever develops due to combined effects of exogenous pyrogens from pathogen and endogenous pyrogens from endotheliopathy. On the other hand, other non-septic (and non-infectious) critical illnesses such as trauma and cancer present with inflammatory fever [62, 63] only via endothelial pathogenesis.

It should be emphasized that in sepsis inflammation is not the cause of endotheliopathy, but is the result of endotheliopathy, which is promoted by activated complement system (Fig. 3). Even though inflammation and VMTD occurs simultaneously in endotheliopathy, inflammation does not sufficiently alter activation of coagulation system or cause microthrombogenesis because they are the result of independent molecular processes, and their interaction is not established at molecular level. Although simultaneous occurrence of inflammation and coagulation in endotheliopathy-associated sepsis seems to be consistent with a high profile crosstalk theory, no persuasive evidence based on hemostatic mechanism has been offered for inflammation promoting microthrombosis to cause “DIC” [11–13], Additionally, it should be noted that inflammation does not occur in AA-VMTD even though platelet-ULVWF complexes are the same microthrombi produced by microthrombogenesis in EA-VMTD. This observation also supports inflammation only occurs in endotheliopathy, and inflammation is not the essential component leading to coagulation/microthrombogenesis.

Different from non-septic critical illnesses, sepsis presents with much severer inflammation, which is perhaps due to increased cytokine expression through additional loop of activated circulating immune cell pathway (e.g., T lymphocyte, macrophage, monocyte, neutrophil, and dendritic cell) suggested by another arrow line in Fig. 3 [64]. This pathway could explain why severe inflammation and SIRS are more common in sepsis than in non-septic critical illnesses [65, 66]. Inflammation provokes inflammatory symptoms in sepsis, but the major endothelial pathogenesis determining the severity and outcome of sepsis is elicited by microthrombosis of EA-VMTD/DIT.

### Consumptive thrombocytopenia in critically ill patients (TCIP)

In sepsis, unexplained thrombocytopenia is a very important early telltale sign that endotheliopathy has occurred and on-going microthrombogenesis is generating consumptive thrombocytopenia (Table 1). Thrombocytopenia has been blamed too often to heparin in the critical care setting [67]. The term “thrombocytopenia in critically ill patients” (TCIP) has been used for etiology-undetermined thrombocytopenia after exclusion of known causes of thrombocytopenia (e.g., heparin-induced, drug and transfusion-associated, “DIC”-associated, bone marrow suppression-caused, hypersplenism-related, idiopathic autoimmune-induced, and others.) [68–70]. Often, it was blamed to multifactorial causes, including consumption associated with thrombin-mediated platelet activation [68]. The significance of mild to moderate TCIP in early stage of sepsis has been commonly downplayed in the care of very sick septic patients because its pathogenesis is not clearly understood and hemorrhagic syndrome is uncommon [1]. Now, it is established TCIP is common and predictable hematologic feature in sepsis-associated coagulopathy as noted in Table 1, occurring as a result of consumptive thrombocytopenia via activated ULVWF path [1–4, 20].

Increasing degree of TCIP is typically associated with advancing VMTD in the septic patient. Since “DIC” is associated with thrombocytopenia, hypofibrinogenemia, prolonged prothrombin time, and activated partial thromboplastin time and positive fibrin degradation products, TCIP has been attributed to consumptive thrombocytopenia as well as consumption of coagulation factors contributing to fibrin clots. However, activated TF path alone does not consume platelets in the formation of fibrin meshes/fibrin clots via the extrinsic coagulation cascade. The above abnormal coagulation profile is also consistent with hepatic coagulopathy. The main differentiating laboratory features are increased FVIII and markedly decreased FVII in EA-VMTD/DIT (i.e., “DIC”)–associated hepatic coagulopathy but markedly decreased FVIII and normal FVII in true DIC (e.g., APL). These coagulation characteristics can serve as very useful laboratory diagnostic features between true DIC associated with APL and “DIC” associated with critical illnesses.

In the critical care setting, significant correlations have been observed between the onset, duration and degree of thrombocytopenia, and severity and outcome of critical illnesses [71, 72]. This observation supports that TCIP is a key hematologic phenotype and plays its important role in the endothelial pathogenesis of sepsis.

### TTP-like syndrome

TTP-like syndrome is the hematologic phenotype of EA-VMTD/DIT characterized by TCIP and MAHA associated with endotheliopathy after exclusion of gene mutation associated TTP (GA-VMTD) and antibody-associated TTP (AA-VMTD). It is an acquired VMTD of non-immune type and develops in sepsis and other critical illnesses [3, 73–77]. TTP-like syndrome is very common but often masked because its pathogenesis is no well appreciated by clinicians [3] and it is often classified and reported in the medical literature as TTP, thrombotic microangiopathy, “DIC”, HUS and unclassifiable microthrombosis. Most commonly, TTP-like syndrome in sepsis has been misdiagnosed as DIC, and the hematologic feature of MAHA is often not uncovered because mild to modest anemia brings very little attention in critically ill patients.

TTP is very similar to hematologic phenotype of EA-VMTD (i.e., TTP-like syndrome) although the pathophysiological mechanism is diametrically different from TTP-like syndrome. TTP occurs due to ADAMTS13 deficiency and typically provokes “microvascular thrombosis” in capillaries and arterioles of the kidneys and brain because microthrombi are formed in microcirculation [78, 79] and become trapped in the microvasculature in situ, promoting activation of “aberrant” ULVWF path of hemostasis without intravascular injury. However, TTP-like syndrome (e.g., sepsis-associated coagulopathy) occurs due to microthrombi that are anchored to ECs of smaller and larger vasculatures as well as capillaries and arterioles and provokes “vascular microthrombosis”, promoting activation of “normal” ULVWF path of hemostasis due to endothelial injury.

Unlike TTP that creates higher stress force when red cells pass through severely occluded microvascular space of the bloodstream [80], perhaps microthrombi strings of EA-VMTD anchored to ECs that create milder shear stress in smaller and larger vasculatures and produce fewer schistocytes that lead to mild MAHA, resulting in less apparent TTP-like syndrome. Milder shear stress is the likely reason why schistocytes are rare and hemolytic anemia is more covert in sepsis. Thus, MAHA is less commonly unmasked, but hemolytic anemia can be an important clue establishing the sepsis-associated VMTD if an experienced hematologist carefully evaluates the thrombocytopenic patient in sepsis with a high index of suspicion. This will save many lives if the diagnosis of TTP-like syndrome can be uncovered in the earliest stage of sepsis. In regard to shear stress, exception may be hemolytic-uremic syndrome associated with EA-VMTD, in which schistocytes are encountered in a large number and produces severe MAHA. It could due to rich microvasculatures present within the kidneys.

**Fig. 4:**
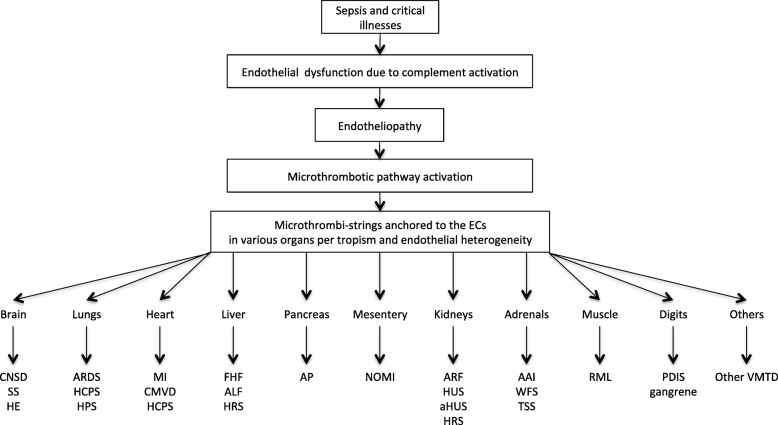
Pathogenesis of multiorgan dysfunction syndrome in sepsis-associated vascular microthrombotic disease. The pathogenesis of MODS in sepsis is summarized. Any organ can be involved by VMTD. However, MODS is much more common in vital organs such as the lungs with ARDS, the brain with CNSD, and the kidneys with acute renal failure. The illustration is self-explanatory. Abbreviations: *AAI* acute adrenal insufficiency, *ALF* acute liver failure, *aHUS* atypical hemolytic uremic syndrome, *AP* acute pancreatitis, *ARDS* acute respiratory distress syndrome, *ARF*, acute renal failure, *CNSD* central nervous system dysfunction, *CMVD* coronary microvascular disease, *FHF* fulminant hepatic failure, *HCPS* hantavirus cardio-pulmonary syndrome, *HE* hepatic encephalopathy, *HPS* hantavirus pulmonary syndrome, *HRS* hepato-renal syndrome, *HUS* hemolytic-uremic syndrome, *NOMI* non-occlusive mesenteric ischemia syndrome, *PDIS* peripheral digit ischemic syndrome, *RML* rhabdomyolysis, *SS*, stroke syndrome, *TSS* toxic and septic shock syndrome, *WFS* Waterhouse-Friderichsen syndrome

Even though both TTP-like syndrome and TTP are pathologically characterized by DIT and clinically by VMTD, the genesis and clinical features between two different types of VMTD [1–4] are very different as presented in Table 2. Variable phenotypic features are the reason why TTP-like syndrome can be easily overlooked when it occurs as a result of EA-VMTD in sepsis and critical illnesses [1–3, 19, 20, 76, 77]. This is particularly true in sepsis, trauma, postoperative TTP [81], and cancer [82, 83]. Because microthrombosis may occur in both smaller and larger vasculatures of various organs in EA-VMTD [84, 85], exotic and fanciful phenotypes are not uncommon as shown in Fig. 4 [3].

**Table 2:** Pathogenetic difference of VMTD between TTP and sepsis-associated TTP-like syndrome

	TTP (GA-VMTD and AA-VMTD)	TTP-like syndrome in sepsis (EA-VMTD)
Primary event	Hereditary TTP (GA-VMTD)	Sepsis due to pathogens
	(due to mutation of ADAMTSD13 gene)	(e.g., bacteria; viruses; fungi; rickettsia; parasites)
	Acquired TTP (AA-VMTD)	↓
	(due to anti-ADAMTS13 antibody)	Complement activation → MAC (C5b-9) formation (?)
	↓	↓
Secondary event	Excess of circulating ULVWF & platelets	C5b-9-induced endotheliopathy
	↓	↓
	Microthrombogenesis, leading to ULVWF-platelet complex formation in microcirculation	ULVWF released from ECs & anchored to endothelial membrane to recruit platelets → microthrombogenesis
	↓	↓
Tertiary event	Microthrombi in microvasculatures	Microthrombi strings in smaller and larger vasculatures
	↓	↓
Final event	Microvascular thrombosis	Vascular microthrombosis
	↓	↓
	GA-VMTD; AA-VMTD	EA-VMTD
	↓	↓
	TTP	TTP-like syndrome

*Abbreviations*: *ECs* endothelial cells, *MAC* membrane attack complex, *TTP* thrombotic thrombocytopenic purpura, *VMTD* vascular microhrombotic disease, *AA-VMTD* antibody-associated VMTD, *EA-VMTD* endotheliopathy-associated VMTD, *ULVWF* unusually large von Willebrand factor multimers

Unexplained thrombocytopenia and hemolytic anemia could be the first clue of serious sepsis, which should be evaluated immediately to determine any evidence of mild MAHA and TTP-like syndrome [86–89]. If a patient presents with minimal number of schistocytes [80–83, 88, 89] and TCIP in sepsis, MODS might be developing in unusual organs such as the lungs, heart, pancreas, adrenals, digits, liver, muscles and others as well as the brain and kidneys [50, 90–93]. In those cases, the evidence of hemolysis with reticulocytosis, hypohaptoglobinemia, indirect hyperbilirubinemia and elevated lactic acid dehydrogenase [1–4] should confirm the diagnosis of TTP-like syndrome as well as EA-VMTD/DIT.

### Multiorgan dysfunction syndrome (MODS)

Mortality of sepsis is highly correlated to the development of MODS. The pathogenetic mechanism of MODS is circulatory dysfunction caused by vascular microthrombosis, obstructing blood flow and decreasing oxygenation in various organs [1–3, 33]. Further progression to hypoxia would lead to organ failure and hasten the demise of the patient.

The mechanism developing different organ phenotypes of MODS in EA-VMTD is the result of complex interaction involving endothelial heterogeneity of the host and tropism of the pathogen. Endothelial heterogeneity is governed by endowed molecules in the endothelium and tropism in different types of pathogen determines the invasiveness of pathogen in each patient. Unique expressivity in the host endothelium and specific affinity of the pathogen to different host organs appears to create variable organ syndromes. Figure 4 summarizes the examples of MODS in various organs that had been known to occur in EA-VMTD. The organ examples showing affinity to pathogens could be the brain with encephalopathy [94] due to various encephalitis viruses, lungs with acute respiratory distress syndrome (ARDS) [70] due to severe acute respiratory syndrome (SARS)-CoV or middle east respiratory syndrome (MERS)-CoV, kidneys with hemolytic-uremic syndrome (HUS) [29] due to STEC, and others.

Currently the pathophysiological mechanism of organ dysfunction is thought to be the results of the direct invasion of pathogen into the tissue of the involved organs. This mechanism may lead to combined compromise of the endothelium and SET/EVT, triggering cellulitis or abscess and macrothrombsis. However, this endothelial breach occurs uncommonly because although sepsis-induced endotheliopathy is universal, typically SET/EVT is not compromised in sepsis. MODS occur as a result of microthrombosis that causes reversible hypoxia in organs unless it advances to terminal multiorgan failure. Organ dysfunction syndrome in sepsis, including fulminant hepatic failure (FHF)/acute liver failure [92], acute necrotizing pancreatitis [93], myocardial infarction [95], acute adrenal insufficiency [50, 96, 97], and rhabdomyolysis [98] may be reversible with improvement of sepsis unless necrotic and gangrenous change develops as seen in tissue gangrene [90] and peripheral digit ischemic syndrome [91]. More serious combined phenotypic syndromes, which underlying pathogenesis is still being debated, include hepatic encephalopathy [92], hepato-renal syndrome [99, 100], hepatic coagulopathy [1–3, 101], and cardio-pulmonary syndrome [102]. Clinicians should take the advantage of hemostatic nature of VMTD when therapeutic approach is considered.

Since the phenotypes of MODS are clinical syndromes occurring due to vascular microthrombosis, it should be stressed that ARDS, HUS, FHF, rhabdomyolysis or acute pancreatitis does not cause sepsis-associated coagulopathy or endotheliopathy. Instead, these organ phenotypes are the manifestations of sepsis-associated coagulopathy that develop due to complement-induced endotheliopathy and subsequent microthrombogenesis. For example, case reports in medical literature have often cited that pancreatitis was responsible for TTP-like syndrome [93] and caused acute renal failure [47], encephalopathy [48] and FHF [49]. These articles should have stated that TTP-like syndrome (i.e., EA-VMTD) caused pancreatitis as well as other organ dysfunction.

### Septic shock due to adrenal insufficiency

Septic shock is a serious and well-known sepsis-associated syndrome, presenting with altered mental state and hypotension associated with hyperthermia/hypothermia, tachycardia/tachypnea, and oliguria. Precipitating circulatory failure with hypotension suggests it is an advancing state of multiple vital organ failure. Therefore, septic shock had been ascribed to underlying circulatory and cellular metabolic abnormalities profound enough to substantially increase mortality [103]. Although multiple organ dysfunctions take place due to hypoxia induced by DIT, the pathophysiological mechanism of septic shock is still an unsolved riddle. There are no answers to the questions on how hypotension is incited by DIT and what kinds of cellular metabolic abnormalities develop in septic shock.

**Fig. 5:**
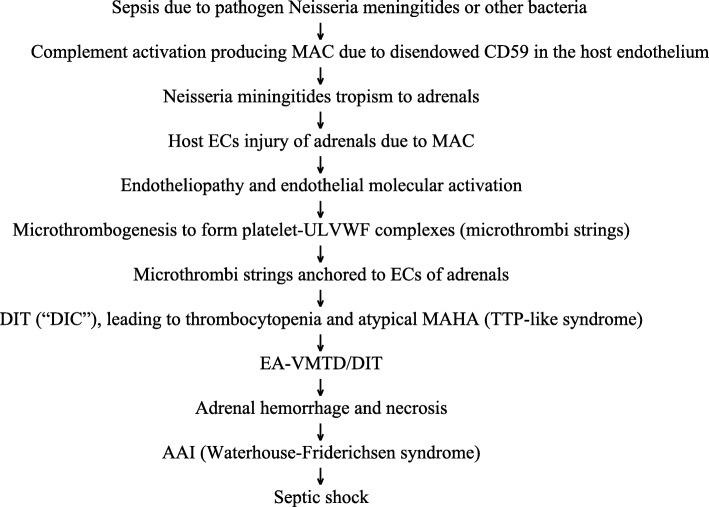
Pathogenesis of septic shock: acute adrenal insufficiency model. Abbreviations: *AAI* acute adrenal insufficiency, *DIT* disseminated intravascular microthrombosis, *ECs* endothelial cells, *MAC* membrane attack complex, *MAHA* microangiopathic hemolytic anemia, *MODS* multiorgan dysfunction syndrome, *TTP* thrombotic thrombocytopenic purpura, *ULVWF* unusually large von Willebrand factor multimers

According to “two-activation theory of the endothelium”, microthrombi strings anchored to ECs within the vasculature trigger circulatory dysfunction, but it is difficult to incriminate the onset of hypotension and “shock” to hypoxia, inflammation or yet unidentified metabolic molecules. However, enough evidences have been accumulated that septic shock represents adrenal crisis/acute adrenal insufficiency (AAI) associated with adrenal hemorrhage and/or necrosis due to DIT. AAI is a more serious form of MODS [104–106]. For example, the tropism of pathogen Neisseria meningitides to the adrenal glands causes Waterhouse-Friderichsen syndrome [50, 96, 97, 106]. Indeed, “DIC”, which presently is being renamed as TTP-like syndrome or EA-VMTD/DIT [1–4, 20], has been associated with Waterhouse-Friderichsen syndrome [96, 97, 106, 107]. The role of AAI in septic shock has been established [107–110]. Its proposed pathogenesis is illustrated in detail in Fig. 5. This proposition of adrenal crisis/AAI resulting from microthrombosis would have a significant ramification in the management of septic shock [111].

### “DIC” and DIT

When we look back to the enigmatic nature of true DIC (i.e., APL-associated coagulopathy), “DIC” (e.g., “DIC”, acute “DIC” and chronic “DIC” in critical illnesses), DIT (e.g., VMTD: TTP of GA-VMTD and AA-VMTD, TTP-like syndrome of EA-VMTD), HUS, thrombotic microangiopathy, their concepts are ambiguous and contradictive one another. Additionally, many of these disorders can occur as a serious life-threatening complication of sepsis or other critical illnesses and are hemostatic diseases presenting as microthrombosis or microthrombo-coagulopathy [20] without clear distinction. This complexity has contributed to the confusion on the exact nature of coagulopathy and thrombopathy in sepsis. In the medical literature, poorly defined sepsis-associated coagulopathy has often been called to be DIC. Sometimes, even proficient coagulation specialists have expressed uneasiness using the term DIC because the diagnosis and pathogenesis could not be unequivocally established because fatality be high and no known effective treatment available. This ambiguity in the concept of DIC must have contributed to the poor outcome of the treatment in sepsis, including unsatisfactory clinical trials and inappropriate anticoagulation, antithrombotic therapy and plasma therapy.

Recently, the concept of “DIC” has been reappraised as DIT, which clinical disease is EA-VMTD/DIT [1–4, 20]. If we move “DIC” from the column of DIC to that of EA-VMTD/DIT, the leftover is true DIC occurring in APL that is coagulopathy associated with hemorrhagic syndrome [2, 3, 20], in which fibrin clots are formed by fibrinogenesis via extrinsic coagulation cascade from activated aberrant TF path [20]. In true DIC associated with APL, the intrinsic character of blood clots is fibrin clots made of fibrin meshes via activated TF path, but in “DIC” of sepsis-associated coagulopathy, the intrinsic character of blood clots is microthrombi composed of platelet-ULVWF strings via activated ULVWF path. Therefore, sepsis-associated coagulopathy is “DIC”, and chronic “DIC” is EA-VMTD/DIT (i.e., TTP-like syndrome). Thus, acute “DIC” is EA-VMTD/DIT with hepatic coagulopathy.

Now, we have hope that effective treatments such as anti-microthrombotic therapy could become available to save lives in sepsis-associated EA-VMTD/DIT. Since “DIC” coined as DIC by Donald McKay [112] in 1950 and with the works of clinicians and coagulation specialists [113–116], the pathophysiological mechanism of “DIC” has been eventually identified to be endotheliopathy-associated microthrombosis. Finally, the analysis of the molecular event of hemostasis in the pathogenesis of EA-VMTD can explain the mechanisms of microvascular thrombosis/vascular microthrombosis, thrombo-hemorrhagic syndrome, abnormal coagulation profile in “DIC”, coexistence of “DIC” and liver disease in acute “DIC”, endothelial contribution to hemostasis, and role of ULVWF path in thrombogenesis. It also explains the meaning of confusing “DIC” markers such as thrombin generation, thrombin-antithrombin complex, tissue factor pathway inhibitor, activated protein C, and thrombomodulin. These markers are not from “DIC” but from secondary events of VMTD such as tissue damage from MODS, vascular accesses during hospitalization, hepatic coagulopathy or combined micro-macrothrombotic syndrome.

The differential features of sepsis-associated “DIC” from other thrombo-coagulopathies are summarized in Table 3. Since “DIC” in sepsis is not coagulation disorder resulting in fibrin clots, but is microthrombotic disorder (DIT) resulting in microthrombi, this paradigm shift from “DIC” to EA-VMTD/DIT has been able to identify the mechanism of microthrombogenesis and true character of microthrombi. While continuing on “DIC” debates to date, nature finally has endowed us the insights that have helped to construct the pathophysiological mechanism of normal hemostasis and thrombogenesis (Fig. 2) [4, 20].

**Table 3:** Differential features of sepsis-associated “DIC” and other thrombo-coagulopathies

	“DIC” without hepatic coagulopathy	“DIC” with hepatic coagulopathy	EA-VMTD/DIT without hepatic coagulopathy	True DIC
Associated disease	Sepsis Other critical illnesses	Sepsis Other critical illnesses FHF	Sepsis Other critical illnesses	APL
Mechanism				
Complement activation	+	+	+	No evidence
Endotheliopathy	+	+	+	No evidence
Inflammatory path	Activated	Activated	Activated	No evidence
Activated hemostatic path	ULVWF	ULVWF	ULVWF	Aberrant TF path
Thrombogenesis	Via micothrombogenesis	Via micothrombogenesis	Via micothrombogenesis	Via fibrinogenesis
Liver involvement	None	FHF	None	Unlikely to occur
Pathology				
Coagulation disorder	VMTD	VMTD with FHF	VMTD	Hemorrhagic disorder
Character of blood clots	Microthrombi	Microthrombi	Microthrmbi	Fibrin clots
Nature of blood clot	Platelet + ULVWF	Platelet +ULVWF	Platelet +ULVWF	Fibrin meshes
Hematology				
Platelet	Decreased	Decreased	Decreased	Decreased due to APL
MAHA	+	+	+	–
FVIII	Normal/increased	Markedly increased	Normal/increased	Markedly decreased
PT/aPTT	Normal	Prolonged	Normal	Prolonged
Fibrinogen	Normal	Decreased	Normal	Decreased
Clinical Phenotype	MODS	MODS	MODS	Hemorrhagic syndrome
	TTP-like syndrome	TTP-like syndrome	TTP-like syndrome	
	Chronic “DIC”	Acute “DIC”	EA-VMTD/DIT	
		FHF		
Correct diagnosis	EA-VMTD/DIT	EA-VMTD/DIT with hepatic coagulopathy	EA-VMTD/DIT	True DIC
Disease designation	EA-VMTD	EA-VMTD with hepatic coagulopathy	EA-VMTD	Consumption coagulopathy

*Abbreviations*: *APL* acute promyelocytic leukemia, *DIC* disseminated intravascular coagulation, *“DIC”* ill-founded DIC, *DIT* disseminated intravascular microthrombosis, *FHF* fulminant hepatic failure, *MAHA* microangiopathic hemolytic anemia, *MODS* multiorgan dysfunction syndrome, *PT* prothrombin time, *aPTT* activated partial thromboplastin time, *TTP* thrombotic thrombocytopenic purpura, *ULVWF* unusually large von Willebrand factor multimers, *EA-VMTD* endotheliopathy-associated vascular microthrombotic disease

^a^Please note that “DIC” without hepatic coagulopathy is exactly the same to EA-VMTD/DIT without hepatic coagulopathy, which is also consistent with the thesis that “DIC” is DIT

### Systemic inflammatory response syndrome (SIRS)

SIRS has been defined as severe febrile and toxic clinical syndrome associated with variable organ dysfunction to the nonspecific but dreadful insult of septic and/or non-septic origin. The clinical description of this syndrome has included severe inflammation and MODS [117–119] associated with various organ phenotypic manifestations due to disseminated microthrombosis and vascular hypoxia such as ARDS, HUS/acute renal failure, and FHF/acute liver failure.

According to the “two-activation theory of the endothelium”, SIRS can be easily recognized in the context of ultimate manifestation of severe inflammation as presented in Fig. 3. SIRS represents intense clinical syndrome due to combined activation of inflammatory pathway, promoting cytokine release and storm, and microthrombotic pathway, triggering platelet activation and endothelial exocytosis, which results in EA-VMTD/DIT. SIRS is more common in severe sepsis compared to non-septic critical illnesses. TCIP occurs often due to platelet consumption and is an early marker of SIRS as in sepsis. Severer thrombocytopenia is more likely associated with combined syndrome of SIRS and MODS [119, 120].

### Combined micro-macrothrombotic syndrome

**Fig. 6:**
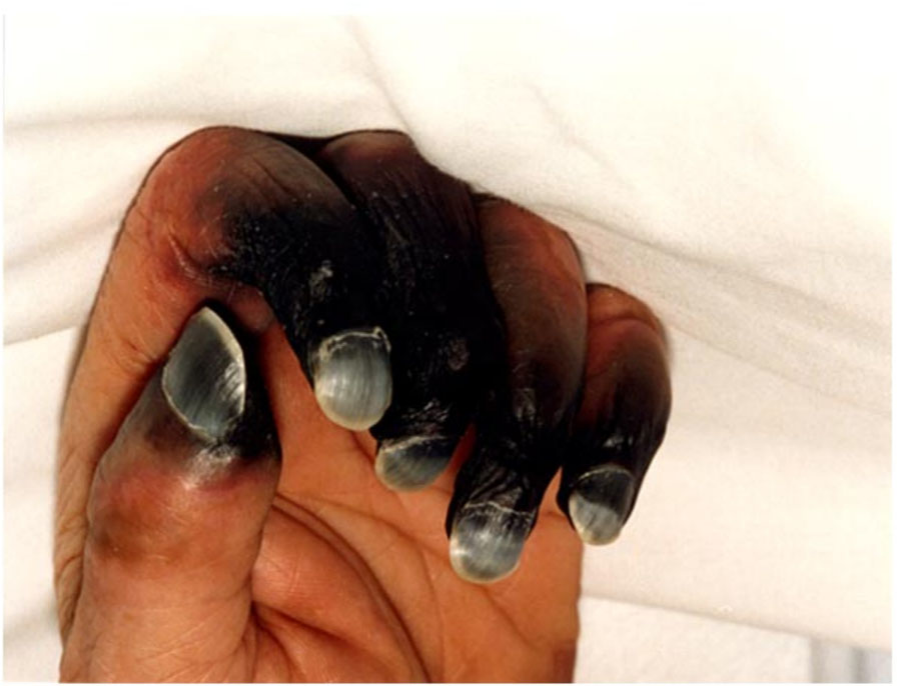
Combined micro-macrothrombotic syndrome in EA-VMTD showing well demarcated symmetrical peripheral gangrene. This photo demonstrates peripheral digit ischemic syndrome in a young man with severe meningococcemia who developed classical “DIC” (TTP-like syndrome). Following a surgical procedure. Severe symmetrical well-demarcated dry gangrenes developed in the fingers of both hands. He survived, but lost gangrenous parts of fingers. This syndrome can be explained by combined micro-macrothrombotic syndrome per “two-path unifying theory” as noted in the text

It is uncommon, but a unique and mysterious thrombosis syndrome often occurring in sepsis with well-demarcated symmetrical peripheral gangrene shown in Fig. 6 [90, 91, 121, 122]. This gangrene developing in “DIC” has been well documented in the literature [91, 123–125], which has been an unexplainable complication of TTP-like syndrome because microthrombosis alone does not cause gangrene. The pathogenesis is unknown, but this author attributes this to combined micro-macrothrombotic syndrome, which becomes obvious if we understand “two-path unifying theory” of hemostasis.

Since EA-VMTD/DIT is composed of platelet-ULVWF complexes via lone activation of ULVWF path, TF path-related thrombin and fibrin markers should not be expressed in EA-VMTD/DIT. However, in sepsis-associated coagulopathy, additional TF path activation may occur due to incidental vascular damage extending from the endothelium to SET/EVT. These may be seen in advanced stage of MODS and hepatic coagulopathy. A more serious condition is multiple macrothrombi associated with gangrene developing in sepsis-associated coagulopathy (EA-VMTD). This disorder typically presents with well-demarcated symmetrical peripheral gangrene involving digits and extremities. This disorder has been termed symmetrical peripheral gangrene [121–125], or peripheral digit ischemic syndrome in the postoperative patient [91]. Also, extensive purpura and ischemic gangrene seen in septic patient typically occurring in thrombophilic neonates (purpura fulminans) is consistent with combined micro-macrothrombotic syndrome. These severe sepsis-associated complex thrombopathic disorders suggest “additional vascular injury” and/or underlying “thrombophilic state” such as protein C deficiency, protein S deficiency, or FV-Leiden play the role in provoking combined micro-macrothrombotic syndrome.

Currently, symmetrical peripheral gangrene in sepsis is suspected to occur due to vasopressor agents used in septic shock. However, a better explanation rests on combined hemostasis of microthrombogenesis and macrothrombogenesis although the latter is secondary event occurring as a complication of EA-VMTD/DIT. The pathophysiological mechanism of combined micro-macrothrombotic syndrome can be easily supported by “two-path unifying theory”. Typically, first set-up is the hospital admission of a patient with sepsis that provokes EA-VMTD/DIT. The patient would need multiple venous and arterial accesses and/or perhaps surgery during hospitalization [91]. This vascular intervention causes intravascular injury, leading to damage of SET/EVT and release of TF. Activated TF path leads to fibrinogenesis and forms fibrin clots in circulation. Since on-going ULVWF path is already active from endotheliopathy and microthrombi strings are being formed, fibrin clots and some of microthrombi would be unified and produce intravascular macrothrombi via macrothrombogenesis [20]. Macrothrombi could travel to the peripheral arterial vasculatures in small and large arteries of the digits and extremities. This combined micro-macrothrombotic syndrome could lead to multiple well-demarcated peripheral symmetrical gangrene at distal vascular trees.

## Therapeutic approaches for sepsis-induced endothelial pathogenesis

Normal protective physiologic immune systems eradicate pathogen in early stage of sepsis with help of prompt use of effective antimicrobial therapy. However, if immune system is or becomes ineffective, sepsis advances to endotheliopathy, orchestrating multiple septic syndromes. If this serious pathogenesis is activated, the phase of sepsis may be shifting from protective role of the immune system to destructive role of the endothelial system.

Although inflammation is a serious clinical syndrome, it is not the main contributor leading to fatality of the host, but microthrombogenesis is the culprit. Since EA-VMTD/DIT and “DIC” are conceptually identical and can be understood best as TTP-like syndrome caused by oversupply of ULVWF from endothelial exocytosis, the term TTP-like syndrome will be used in the discussion of therapeutic modalities in sepsis for the rest of this article.

### Therapeutic plasma exchange

Therapeutic plasma exchange (TPE) has been effective treatment for TTP-like syndrome if the therapy had been promptly applied [76, 78, 81, 89, 91, 126, 127]. Perhaps untimely treatment with TPE due to the delay in establishing the diagnosis has been the main factor for poor outcome of EA-VMTD [78, 81, 89, 91]. The rationale utilizing TPE in TTP-like syndrome is to cleave and/or remove the excess of ULVWF in the patient plasma with replacement of normal donor plasma that contains adequate amounts of ADAMTS13 [19]. Indeed, TPE has been extensively utilized by clinicians when a patient was thought to have atypical TTP, TTP-like syndrome or “thrombotic microangiopathy”. In retrospective review of many reports and case studies, most of them were found to have the clinical features of EA-VMTD/DIT. We know that TPE is effective in TTP-like syndrome, including “DIC” [128], and is being recommended, in patients with thrombocytopenia and MAHA with/without MODS. TPE is a life-saving measure in TTP-like syndrome at this time.

As far as sepsis is concerned, TPE was utilized in the treatment of sepsis in few respectable clinical trials [129, 130] and pilot studies as well as case studies [131–134]. These studies were done even without the understanding of the concept of microthrombogenesis and mechanism of endothelial pathogenesis. Interestingly, TPE seemed to be more effective than plasma filtration therapy in sepsis [135, 136]. Additionally, TPE was also effective for “DIC” [128, 134] and MODS [131, 134]. The response of sepsis to TPE also supports septic syndromes are the manifestations of DIT. TPE is expected to be a very effective therapy if utilized in the earliest possible time in the septic syndromes.

### Anti-complement therapy

Atypical HUS (aHUS) is a well-recognized syndrome associated with “dysfunctional” or more likely with “unprotected” complement activation [22, 29]. The effectiveness of anti-complement therapy is established for aHUS [137, 138]. Since complement activation in sepsis to kill invading pathogen can be inhibited by anti-complement therapy in early immune defense system, theoretically anticomplement therapy such as eculizumab should be contraindicated in sepsis at this time until we know more about the precise role of complement in advancing stage of endotheliopathy. However, potential benefit in non-pathogen related EA-VMTD such as trauma, toxin and envenomation is an interesting speculation.

### Anti-microthrombotic therapy

TPE as a surrogate form of antimicrothrombotic therapy is highly time-sensitive, but is an effective treatment. However, if the treatment is delayed due to technical limitation and/or becomes complicated by vascular volume overload or electrolyte imbalance, the outcome from TPE is likely to be unfavorable in the septic syndromes and other critical illnesses.

Theoretically, the ideal treatment is targeted therapy with one of antimicrothrombotic agents such as recombinant ADAMTS13 (rADAMTS13) at the earliest possible time. The studies have shown ADAMTS13 is effective in cleaving platelet-ULVWF complexes in vitro. Antimicrothrombotic therapy has been advocated in vivo use for potential benefit in acquired TTP (TTP-like syndrome) [139–142]. Recombinant ADAMTS13 is being used only in Phase I study for GA-VMTD (i.e., hereditary TTP) at this time. Since its proteolytic activity on ULVWF is well established, this investigational agent should be available for clinical trials in EA-VMTD, including septic syndromes and “DIC” as well as MODS caused by EA-VMTD. In a latest study on acquired TTP, anti-microthrombotic agent caplacizumab, which is anti-von Willebrand factor (VWF) nanobody [143], has shown significant clinical benefit. This positive result also supports the role of ULVWF path in TTP-like syndrome as well as EA-VMTD/DIT.

Anti-microthrombotic therapy can be utilized with a close monitoring of the platelet count, intravascular hemolysis, and evaluation of organ dysfunctions. Streamlined therapeutic trials with recombinant ADAMTS13 should be proposed for the patients with septic syndromes associated with EA-VMTD/DIT as soon as possible.

## Conclusion

The endothelial pathogenesis of sepsis and septic shock is due to lone activation of ULVWF path without activation of TF path of hemostasis. The molecular pathogenesis of sepsis is clearly established from the dual concepts based on “two-path unifying theory of hemostasis” and “two-activation theory of the endothelium”. In sepsis, complement activation that is destructive to ECs of the host can provoke endotheliopathy, which promotes two independent molecular events, leading to activation of inflammatory pathway and microthrombotic pathway. The result is endotheliopathy associated inflammation and EA-VMTD presenting with TTP-like syndrome. In a big picture, physiologic protective immune system guards the host and eliminates invading pathogen, but pathologic destructive endothelial system is detrimental to the host when protective immune systems are compromised and antibiotics are not optimally effective. With better understanding of sepsis-associated coagulopathy (microthrombopathy), effective treatments could be designed by targeting the microthrombotic pathway of endothelial pathogenesis.

## Abbreviations

“DIC”: Ill-founded disseminated intravascular coagulation
AAI: Acute adrenal insufficiency
AA-VMTD: Antibody-associated VMTD
ADAMTS13: A disintegrin and metalloproteinase with a thrombospondin type 1 motif, member 13
aHUS: Atypical HUS
ALF: Acute liver failure
aMAHA: Atypical MAHA
AP: Acute pancreatitis
APC: Activated protein C
APL: Acute promyelocytic leukemia
ARDS: Acute respiratory distress syndrome
ARF: Acute renal failure
CMVD: Coronary microvascular dysfunction
CNSD: Central nervous system dysfunction
DIC: Disseminated intravascular coagulation
DIT: Disseminated intravascular microthrombosis
DSS: Dengue shock syndrome
EA-VMTD: Endotheliopathy-associated VMTD
ECs: Endothelial cells
EVT: Extravascular tissue
FHF: Fulminant hepatic failure
FVIIa: Activated factor VII
GA-VMTD: Gene mutation-associated VMTD
GE: Gastroenteritis
HCPS: Hantavirus cardiopulmonary syndrome
HE: Hepatic encephalopathy
HFRS: Hemorrhagic fever with renal syndrome
HPS: Hantavirus pulmonary syndrome
HRS: Hepato-renal syndrome
HUS: Hemolytic-uremic syndrome
IF: Interferon
IL: Interleukin
LPS: Lipopolysaccharide
MAC: Membrane attack complex
MAHA: Microangiopathic hemolytic anemia
MI: Myocardial infarction
MODS: Multi-organ dysfunction syndrome
NETs: Neutrophil extracellular traps
NO: Nitric oxide
NOMI: Non-occlusive mestenteric ischemia
PDIS: Peripheral digit ischemic syndrome
rADAMTS13: Recombinant ADAMTS13
RML: Rhabdomyolysis
RMSF: Rocky mountain spotted fever
SET: Subendothelial tissue
SFTS: Severe fever with thrombocytopenia syndrome
SIRS: Systemic inflammatory response syndrome
SS: Stroke syndrome
STEC: Shiga toxin-producing *Escherichia coli*
TCIP: Thrombocytopenia in critically ill patients
TF: Tissue factor
TFPI: Tissue factor pathway inhibitor
TM: Thrombomodulin
TMOF: Thrombocytopenia and multiple organ failure
TNF: Tumor necrosis factor
TPE: Therapeutic plasma exchange
TSS: Toxic shock syndrome
TTP: Thrombotic thrombocytopenic purpura
ULVWF: Unusually large von Willebrand factor
VMTD: Vascular microthrombotic disease
WFS: Waterhouse Friderichsen syndrome

## Author’s information

Board certified hematologist and board certified hematopathologist, retired professor of medicine in the University of California Irvine School of Medicine, and retired professor of medicine in the Wright State University of School of Medicine.
